# Influence of Diet, Dietary Products and Vitamins on Age-Related Cataract Incidence: A Systematic Review

**DOI:** 10.3390/nu15214585

**Published:** 2023-10-28

**Authors:** Martyna Falkowska, Maryla Młynarczyk, Zuzanna Micun, Joanna Konopińska, Katarzyna Socha

**Affiliations:** 1 Department of Bromatology, Faculty of Pharmacy with the Division of Laboratory Medicine, Medical University of Białystok, Mickiewicza 2D, 15-222 Białystok, Poland; katarzyna.socha@umb.edu.pl; 2 Department of Ophthalmology, Medical University of Białystok, M. Skłodowskiej-Curie 24a, 15-276 Białystok, Poland; mromaniuk2121@gmail.com (M.M.); z.micun@wp.pl (Z.M.); joannakonopinska@o2.pl (J.K.)

**Keywords:** cataract, aged-related cataract, ARC, diet, dietary patterns, dietary products, vitamins, minerals, carotenoids

## Abstract

Cataract, defined as the opacification of the lens that prevents clear vision, is a leading cause of vision loss and impairment worldwide. Elderly people comprise the highest proportion of those suffering from this eye disease. According to the National Institute of Health (NIH), the risk of developing aged-related cataract (ARC) increases with every decade of life, starting from the age of 40. Despite progress in surgical treatment methods, life-style modifications may be beneficial in prevention or slowing down the progression of ARC. This systematic review aims to summarize studies on the significance of specific nutritional patterns, dietary products, vitamins, minerals, and carotenoids intake in the onset or progression of ARC. In this context, the presented paper thoroughly analyzes 24 articles, following the PRISMA guidelines. The results indicate significant protective effects of various dietary patterns, including the Korean balanced diet, vegetarian diet, “dairy products and vegetables”, “traditional”, “antioxidant”, and “omega-3” patterns. Additionally, the consumption of fruits, vegetables, legumes, nuts, skimmed yoghurt, fish, coffee, and vitamins has shown positive effects on cataract incidence. Therefore, further research seems to be essential to gain a better understanding of these associations and to create uniform dietary recommendations for both the vulnerable population and ARC patients.

## 1. Introduction

A cataract is an opacification of the lens which obscures the passage of light, causing a significant decrease in visual acuity [1]. Thus, cataracts are the leading cause of vision loss and impairment worldwide [2]. The lens is a crucial optical component of the eye. It is biconvex and anatomically consists of the cortex, nucleus, and lens capsule. In the course of cataract development, various portions of the lens progressively become cloudy, leading to a gradual deterioration in visual acuity. These opacifications can be observed during physical examination. The etiology of a cataract is multifactorial and not fully explained. Senile cataract (age-related cataract—ARC) is the most common type.

Lens opacities that develop gradually with age, and unlike other types of cataracts, without any specific cause, are known as senile cataracts. Apart from ARC, as regards their etiology, we can distinguish, among others: post-traumatic cataracts and drug-induced cataracts. Cataracts are classified mainly in terms of the location and intensity of lens opacities (The Lens Opacities Classification System III) [3]. The primary locations include the cortex, nucleus, or posterior subcapsular area of the lens [4,5].

In 2020, cataracts caused blindness in 15.2 million people over the age of 50 (with a range of 12.7–17.9 million), while an additional 78.8 million individuals (with a range of 67.2–91.4 million) experienced moderate-to-severe visual impairment (MSVI) [6]. Despite the downward trend in the incidence of blindness caused by both cataract and overall causes [7,8], statistical analysis reveals a 29.7% increase in the absolute number of cataract cases over the last twenty years [6]. The formation of cataracts, although not fully understood, is strongly linked to certain factors: the age of patients, their exposure to ultraviolet radiation, nicotine consumption, and the presence of comorbidities, such as diabetes, hypertension, and chronic kidney disease [9], as well as the use of certain medications [10,11,12]. Given the highest prevalence of specific types of senile cataracts, this review focuses on ARC, which can affect the following lens structures: nucleus, cortex, and posterior subcapsular area.

Pharmacological treatment for cataracts remains an area of active research among many scientists. Currently, there are a limited number of substances on the pharmaceutical market with regulatory approvals that classify them as anti-cataract drugs. Among these are pyrenoxine, which has an inhibitory effect on quinone radicals [13], and potassium iodide, known for its properties that support vitreous body metabolism, thereby preventing the formation of lens opacifications [14]. These substances, along with pupillary dilatation or the use of refractive eyeglasses, can improve the quality of life for patients with early-stage cataracts. Nonetheless, they do not represent the gold standard for managing advanced-stage cataracts, unlike surgical phacoemulsification [15].

Despite the continuously improving surgical techniques for cataract removal, prevention and life-style modification can play a significant role in slowing the progression of this condition. An imbalanced diet may indirectly impact the incidence and severity of cataract progression. Therefore, it is worth considering the influence of food, particularly the presence of antioxidant substances delivered with one’s diet, as well as appropriate choice of nutrients. This approach could be beneficial as a complement to treatment or in inhibiting the progression of the disease [16,17,18].

While there are some reports on the role of diet in the prevalence of ARC, only a few studies focus on more than one nutritional factor. The paucity of research that comprehensively addresses all the dietary elements contributes to the knowledge gap in the field of nutritional prevention of this disease. This gap hinders efforts to inhibit its development and reduce its incidence among the vulnerable population. Moreover, limitations in the size and diversity of areas where population-based studies of diet–ARC relationships have been conducted, as well as the characteristics of the surveyed population, underscore the need for a systematic review summarizing the findings collected to date. The prevalence of ARC and the diversity of dietary patterns across different geographic locations should also receive more careful consideration in available research papers.

The objective of this study was to provide a comprehensive summary of the evidence regarding the links between dietary patterns, consumption of specific food groups, antioxidant vitamins, carotenoids, and certain minerals and the risk of ARC. By understanding the roles of these nutrients in metabolism and cell transport, and their involvement in the development and progression of ARC, we aim to drive advancements in the management and prevention of this condition. In addition, it is essential to emphasize the potential application of the findings from the cited studies in shaping dietary guidelines for susceptible populations and ARC patients.

## 2. Materials and Methods

### 2.1. Search Strategy and Eligibility Criteria

The presented systematic review was prepared in accordance with the Preferred Reporting Items for Systematic Reviews and Meta-Analyses (PRISMA) guidelines [19]. The investigation was conducted in October 2022 and updated with studies published up to September 2023. The following scientific databases were used to search for relevant literature: PubMed, Cochrane Library, Web of Science, and Scopus. The terms utilized to perform the retrieval process were as follows: “cataract” or “age-related cataract” or “ARC” and “diet” or “nutrition”. In addition, further searches were complemented with the phrases “cataract” or “age-related cataract” or “ARC” and “diet products”, “cataract” or “age-related cataract” or “ARC” and “antioxidants”, “cataract” or “age-related cataract” or “ARC” and “vitamins”, “cataract” or “age-related cataract” or “ARC” and “carotenoids”, “cataract” or “age-related cataract” or “ARC” and “minerals”. Furthermore, reference papers from articles extracted in this process were manually reviewed to expand the search.

To determine whether the studies should be included, the following inclusion criteria were used: humans, females, males, adult-aged, cataract, ARC, publication date: up to 10 years earlier, English language, non-animal studies, non-cell studies and non-case reports. In order to reflect the best representation of the impact of food on the incidence of cataracts in the human population, both men and women were included in the systematic review. Furthermore, the appropriate age of the study participants included in this research was essential, due to its association with the prevalence of the condition in question. Limiting the publication time of the studies included in the review was intended to allow the reviewer to focus on the latest scientific findings in the field under review. As for the rejection of studies conducted on animal models, cells or individual case reports, this was to serve to enhance clinical value and facilitate the reference of studies to the entire exposed population.

**Figure 1:**
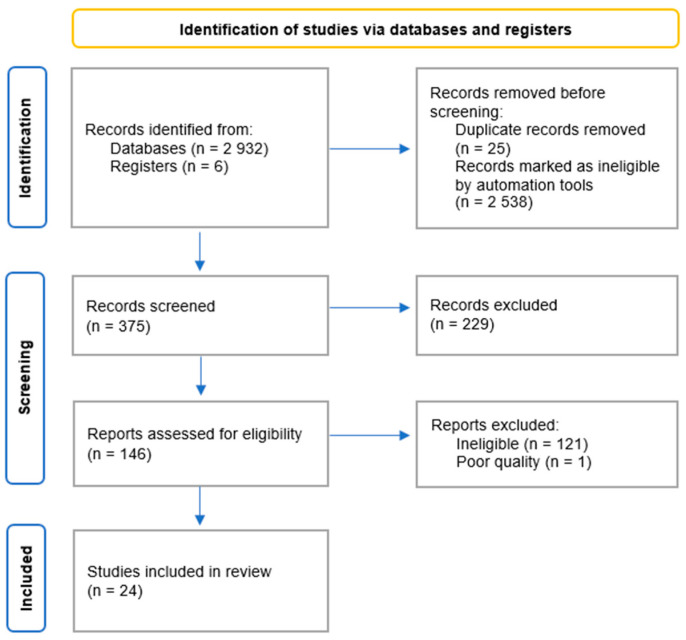
Flow diagram for systematic review.

The search initially yielded 2938 records. After removing duplicates, the number was reduced to 2913. Applying the selection criteria, 2538 records were marked as ineligible by automation tools. After screening the titles and abstracts, 146 records were identified as potentially of interest and subjected to full-text assessment. This led to the exclusion of 121 records. Of the remaining 25 articles, 24 were included in the systematic review following a qualitative assessment. The results of the search process are presented in Figure 1.

The characteristics of the included studies are summarized in Table 1. Nine of them were cohort studies, five were meta-analyses, four were case–control studies, three were cross-sectional studies, and three were controlled intervention studies. None of the authors declared any conflict of interest.

### 2.2. Data Extraction and Assessment of Study Quality

Data extracted from articles were recorded on an MS Word worksheet. These data included the baseline characteristics of the incorporated studies (author, year, and study design), the study group (number of participants, their age, and type of ARC), and key results obtained from them. The assessment of the reports was conducted using the following tools: “Quality Assessment Tool for Observational Cohort and Cross-Sectional Studies”, “Quality Assessment of Systematic Reviews and Meta-Analyses”, “Quality Assessment of Case–Control Studies”, and “Quality Assessment of Controlled Intervention Studies” [44]. These multi-question questionnaires were developed in 2013 by NHLBI as a set of quality assessment tools. Their primary aim was to support reviewers in focusing on concepts central to a study’s internal validity. These tools were specific to certain study designs and had been tested for potential defects in their methodology or implementation. Moreover, they were created for use during the systematic evidence review process to update existing clinical guidelines. The questions used in these assessment tools primarily focused on the research question, the study population, the diagnostic criteria, and the risk of bias. Each question could be answered with the following options: “yes”, “no” or “other” (with “c/d” for “cannot determine”, “n/a” for “not applicable”, and “n/r” for “not reported”), associated with a score of either 1 or 0 points. Following the scoring, each study was divided into one of three quality categories: “good”, “fair” or “poor”. The “good” quality category applied to observational cohort and cross-sectional studies, as well as controlled intervention studies that scored at least 10 points, systematic reviews and meta-analyses with a rating of 6 points and more, and case–control studies with a minimum score of 9 points. The “fair” category included observational cohort and cross-sectional studies, as well as controlled intervention studies scoring at least 5 points, systematic reviews and meta-analyses with a rating of at least 3 points, and case–control studies with a minimum score of 4 points. Research which did not meet the above requirements, by scoring lower than the specified number of points in the quality assessment tests, was classified as “poor”. Only studies with a “good” or “fair” quality score were included in the presented review (Supplementary Tables S1–S4).

The process of data extraction was conducted independently by one of the authors. Any uncertainties in the assessment were resolved through consensus with the other contributors.

## 3. Results

### 3.1. ARC and Different Types of Diets

#### 3.1.1. Mediterranean Diet (MedDiet) 

The MedDiet is defined as a dietary pattern followed by people living along the shores of the Mediterranean Sea. It is widely considered one of the healthiest nutritional models in the world. The diet is essentially plant-based, emphasizing whole, minimally-processed foods, such as green leafy vegetables, fresh fruits, whole grains, nuts, and olive oil. It also involves reducing the consumption of dairy products, seafood, meat, and wine, while allowing only a small amount of sweets and eggs [45,46].

The association between the MedDiet and the risk of cataract surgery was assessed in a 2017 study by Gracia-Layana et al.. Their research, which was part of a large parallel group-randomized trial called PREDIMED, analyzed three nutritional models: (1) the MedDiet supplemented with Extra Virgin Olive Oil (MedDiet + EVOO); (2) the MedDiet supplemented with mixed nuts (MedDiet + Nuts); and (3) a control diet (a low-fat diet according to the American Heart Association guidelines). Despite the significant contribution of the MedDiet to reducing cardiovascular events, this particular study did not show a significant difference in the incidence of cataract surgery between the participants assigned to the MedDiet + EVOO, MedDiet + Nuts and the control groups. The rates observed in this research for the MedDiet + EVOO, MedDiet + Nuts, and the control groups (per 1000 person-years) were 16.9, 17.6, and 16.2, respectively, indicating no meaningful difference between the analyzed groups [20].

#### 3.1.2. Traditional Korean Balanced Diet

The Korean diet (K-diet) is primarily a calorie-restricted and low-in-animal-fats nutritional pattern based on vegetables, grains, legumes, and fish. According to the Seoul Declaration, the definition of the K-diet is as follows: it is rich in cooked rice, kimchi, seasoned vegetables, and medicinal herbs, moderate in fish and pulses, and low in red meat [47]. In the traditional Korean balanced diet, grains, especially rice and barley, are the main sources of carbohydrates, legumes and fish supply protein, and vegetable oils, such as sesame and perilla oils, provide fats [48].

A large-scale hospital-based cohort study conducted by Jee and Park evaluated the association between lifestyle-related risk factors, metabolic syndrome (MS) and ARC. The dietary patterns considered were: the traditional Korean balanced diet (KBD), the Western diet (WD), and a rice-based diet. The results showed that the prevalence of ARC was 20% lower in MS participants with high consumption of the KBD (OR = 0.801, 95%CI = 0.696–0.999, *p* < 0.01) in comparison to those with low consumption. This was the only dietary pattern which demonstrated a significant inverse association with ARC risk in the MS groups. The Western diet and rice-based diet showed no association with ARC risk in either population, whether they had MS or not [21].

#### 3.1.3. Traditional and Dairy-Products-and-Vegetables Dietary Patterns

A comparison of the prevalence of ARC in those following a traditional dietary pattern and a vegetable pattern was presented in the 2021 Amini et al. study. In this paper, a dietary pattern called “dairy products and vegetables” was described as a nutritional model rich in milk products, vegetables, tea, and fats, and low in salt and spices. The “traditional pattern” was distinguished by the consumption of lamb, mutton, and beef, as well as the presence of fats and low intake of white meat, legumes, bread, rice, and other grains. The “carbohydrate and simple sugar pattern” involved consuming high amounts of fresh and processed fruits, vegetables, and sugar, with a low amount of nuts and seeds. Meanwhile, the pattern called “nuts, seeds and simple sugar” was primarily comprised of nuts and seeds, along with sugar. The results of this study showed a protective role for the “dairy products and vegetables” and “traditional” patterns against ARC (OR = 0.301, 95%CI = 0.137–0.658, *p* = 0.002 and OR = 0.393, 95%CI = 0.184–0.842, *p* = 0.036, respectively). In contrast, the “carbohydrate and simple sugar pattern” was associated with an increased likelihood of developing ARC (OR = 5.067, 95%CI = 2.265–11.335, *p* < 0.001) [22].

#### 3.1.4. Vegetarian Diet

The vegetarian diet is a predominantly plant-based dietary pattern, which, depending on the type, may exclude some or all animal-based products [49]. 

In a study conducted by Chiu et al., vegetarians were defined as individuals who had not consumed meat or fish for at least one month before the start of the survey. The main aim of this prospective cohort research was to investigate the association between the vegetarian diet and the risk of ARC. The results showed that the vegetarian diet, in comparison to a non-vegetarian nutritional pattern, contained higher amounts of vegetables (especially soy and nuts), whole grains, dietary fiber, folate, and vitamins A and C, but was not significantly different in terms of the consumption of fruit, processed grains, or supplements. Moreover, after adjustment for demographic, lifestyle, and health factors, such as hypertension, diabetes, hyperlipidemia, corticosteroid prescription, and body mass index, the Taiwanese vegetarian diet proved to be statistically significantly associated with a 20% decreased risk of ARC (HR = 0.80, 95%CI = 0.65–0.99; *p* = 0.04). This association was even more noticeable among overweight participants (BMI ≥ 24 in Taiwan), with an HR = 0.70, 95%CI = 0.50–0.99, and *p* = 0.04 [23].

#### 3.1.5. Nutrient Patterns

In 2017, Sedaghat et al. evaluated the association between dietary patterns and ARC risk. They focused on five different nutrient patterns: “sodium”, “fatty acid”, “mixed”, “antioxidant” and “omega-3”. The “sodium pattern” was characterized by high consumption of sodium, zinc, vitamins B1, B2, and B6, protein, and carbohydrates. The “fatty acid pattern” was primarily correlated with oleic, linoleic, linolenic, and trans fatty acids, as well as monounsaturated, polyunsaturated, and saturated fats, and vitamin E intake. The “mixed pattern” referred to high consumption of calcium, cholesterol, vitamin D, and B12. The “antioxidant pattern” was high in vitamin A, vitamin C, alpha and beta-carotene, whilst the “omega-3 pattern” was rich in eicosapentaenoic acid (EPA) and docosahexaenoic acid (DHA). The result showed that the “sodium pattern” and the “fatty acid pattern” tended to increase the probability of ARC incidence (OR = 1.97, 95%CI = 1.09–3.96, *p* = 0.051 and OR = 1.94, 95%CI = 1.1–3.86, *p* = 0.052, respectively). The “antioxidant pattern” and the “omega-3 pattern” were associated with a significantly lower risk of ARC (OR = 0.21, 95%CI = 0.11–0.40, *p* < 0.001 and OR = 0.71, 95%CI = 0.40–0.92, *p* = 0.04, respectively). No significant association was observed between the “mixed pattern” and ARC (*p* = 0.28) [24].

#### 3.1.6. Pro-Inflammatory Diet

The Dietary Inflammatory Index (DII) is a scoring tool developed to classify diets with regard to their inflammatory potential by evaluating the correlation between nutritional components and six inflammatory markers (IL-1, IL-4, IL-6, IL-10, TNF-α, and CRP). This evaluation algorithm was used, among others, by Shivappa and team to assess the association between diet and cataracts. The results presented in the study showed that the prevalence of ARC was indeed higher in participants with a higher dietary inflammatory potential, both with and without energy adjustment calculations (OR =  2.69; 95%CI =  1.32–2.26, *p* =  0.002 and OR =  1.51; 95%CI  =  1.13–2.03, *p* = 0.006, respectively) [25].

### 3.2. ARC and Different Types of Dietary Products

#### 3.2.1. Fruit and Vegetables

The association between fruit and vegetable consumption and ARC risk was assessed by Pastor-Valero in 2013, as well as by Theodoropoulou et al. in 2014 and Adachi et al. in 2021. These researchers investigated the health status and eating habits of residents of Spain, Greece, and Japan, respectively.

The results of Pastor-Valero’s cross-sectional population-based study initially showed a statistically significant difference between the consumption of combined fruit and vegetables in participants with ARC compared to those without it (444 g/day vs. 488 g/day, *p* = 0.001, respectively). Additionally, the highest versus the lowest intake of combined fruit and vegetables was negatively associated with the prevalence of ARC (OR_adjusted for age, sex, and energy_ = 0.44, 95%CI = 0.25–0.79, *p* = 0.016). After further adjustments, an analogous association was observed between increased consumption of fruit and vegetables (OR_adjusted for marital status, smoking, alcohol consumption, physical activity, supplement use, obesity, and history of diabetes_ = 0.38, 95%CI = 0.20–0.70, *p* = 0.008) [26].

Theodoropoulou et al. found a significant negative association between the incidence of ARC, including all types of cataracts, as well as nuclear cataracts and PSC cataracts, and the consumption of vegetables (OR_cataract overall_ = 0.47, *p* < 0.001; OR_nuclear cataract_ = 0.46, *p* < 0.001; OR_PSC cataract_ = 0.33, *p* < 0.001), fruit (OR_cataract overall_ = 0.53, *p* < 0.001; OR_nuclear cataract_ = 0.51, *p* < 0.001; OR_PSC cataract_ = 0.40, *p* = 0.001), and potatoes (OR _cataract overall_ = 0.76, *p* = 0.004 for cataract overall; OR_nuclear cataract_ = 0.75, *p* = 0.012; OR_PSC cataract_ = 0.59, *p* = 0.004). For cortical cataracts, statistically significant associations were observed only in terms of fruit (OR = 0.33, *p* = 0.01). After mutual adjustment of fruit and vegetable consumption, the associations for cataract overall, nuclear cataract, and PSC cataract remained significant (OR = 0.61, *p* = 0.001, OR = 0.49, *p* < 0.001; OR = 0.48, *p* < 0.001, OR = 0.59, *p* = 0.003; OR = 0.34, *p* < 0.001, OR = 0.50, *p* = 0.008, respectively). Moreover, the intake of citrus and non-citrus fruit proved to be significantly negatively associated with the prevalence of ARC (OR = 0.78, *p* = 0.030; OR = 0.58, *p* < 0.001, respectively) [27].

The large Japanese cohort study conducted by Adachi et al. focused on the consumption of total vegetables, cruciferous vegetables, green and yellow vegetables, and also fruit. The results showed that male participants who consumed the highest amount of total vegetables and cruciferous vegetables, rather than the lowest, had a 23% and 26% lower (OR_Q5 vs Q1 for total vegetables_ = 0.77, 95%CI = 0.59–1.01, *p* = 0.03; OR_Q5 vs Q1cruciferous vegetables_ = 0.74, 95%CI = 0.57–0.96, *p* = 0.02) prevalence of ARC, respectively. Furthermore, the highest versus the lowest intake of total vegetables and cruciferous vegetables was associated with a 33% and 29% decreased prevalence of ARC in male smokers compared with the total male population (OR = 0.67, 95%CI = 0.48–0.96, *p* = 0.01; OR = 0.71, 95%CI = 0.50–1.00, *p* = 0.04, respectively). Moreover, that association was even greater in the group of men over 60 years old compared to younger men (OR_Q5 vs Q1 for total vegetables_ = 0.66, 95%CI = 0.48–0.92, *p* = 0.04; OR_Q5 vs Q1cruciferous vegetables_ = 0.63, 95%CI = 0.46–0.85, *p* = 0.007). In the group of female participants, the opposite result was noticed. The higher the total consumption of vegetables, the higher the prevalence of ARC (OR_Q5 vs Q1 for total vegetables_ = 1.28, 95%CI = 1.06–1.53, *p* = 0.01). A multivariate OR for the highest versus the lowest intake of total vegetables showed a 27% prevalence of ARC (95%CI = 1.06–1.54, *p* = 0.008) in non-smoking women. No significant association between green-and-yellow-vegetable and fruit consumption and ARC was observed in either sex [29].

A study summarizing findings on the association between vegetable consumption and ARC risk was presented in 2015 by Huang et al.. In their meta-analysis, the pooled result showed that the highest vegetable intake, compared to the lowest, was significantly negatively associated with the risk of ARC (RR_summary_ = 0.723, 95%CI = 0.594–0.879, I^2^ = 72.8%). In a stratified analysis by research design, relationships were also discovered in case–control studies and cohort studies (RR_summary_ = 0.583, 95%CI = 0.420–0.809 and RR_summary_ = 0.871, 95%CI = 0.791–0.959, respectively). The highest versus the lowest vegetable consumption was significantly related to the risk of ARC in America and Europe (RR_summary_ = 0.872, 95%CI = 0.791–0.960 and RR_summary_ = 0.507, 95%CI = 0.416–0.619, respectively), but not in other regions of the world [28].

#### 3.2.2. Dairy Products

The study conducted by Camacho-Barcia et al. investigated the association between the consumption of both total and specific types of dairy products (total, whole and skimmed milk, total, whole and skimmed yogurt, and cheese) and the risk of ARC. After an over-5-year follow-up, the results showed that participants with higher intakes of skimmed yogurt had an inverse association with the risk of cataract surgery (second tertile HR_after adjusting for potential confounders_ = 0.62, 95%CI = 0.52–0.74; third tertile HR_after adjusting for potential confounders_ = 0.71, 95%CI = 0.60–0.85, *p* = 0.001). The second tertile of total yogurt intake also indicated a negative association with cataract risk (HR = 0.75; 95%CI = 0.62–0.90, *p* = 0.008). However, these were the only analyzed dairy products that exhibited a protective effect against ARC [30]. 

This result was partially validated by the previously mentioned Amini et al. research, where the “dairy products and vegetables” dietary pattern had a negative association with ARC incidence (OR = 0.301, 95%CI = 0.137–0.658, *p* = 0.002) [22].

#### 3.2.3. Wholegrain and Legume Foods

No significant association between total wholegrain intake and any type of ARC incidence was found in the Australian cohort study conducted by Tan et al.. Furthermore, the analysis of the consumption of brown rice, breakfast cereal, wholemeal/multigrain bread, or oatmeal also showed no statistically significant association with the prevalence of ARC. The highest intake of legumes versus the lowest exhibited a protective effect on PSC cataracts (OR_Q5 v. Q1_ = 0.37, 95%CI = 0.15–0.92, *p* = 0.08). However, there was no significant trend in this association. No associations were observed between pulse consumption and the incidence of cortical or nuclear cataract [31]. Similarly, in the study by Theodoropoulou et al., higher legume and nut consumption, when compared to lower consumption, suggests a trend towards a decrease in the incidence of PSC and cataracts overall (OR = 0.69, *p* = 0.093; OR = 0.84, *p* = 0.075, respectively) [27].

#### 3.2.4. Coffee

In the study conducted by Jee et al., researchers aimed to identify an indirect association between lifestyle and the prevalence of ARC among over 40,000 participants. They examined the correlation between diet and polygenetic risk scores (PRS), which was intended to reflect the genetic influence on ARC risk. The participants were grouped based on higher and lower nutrient consumption. The study found that higher coffee consumption, in comparison to lower consumption, was significantly associated with a reduced ARC prevalence. Furthermore, the incidence of ARC was observed to increase progressively from low-PRS, through medium-PRS, to high-PRS. This suggests that consuming more than 3 g of coffee per day may offer protection against ARC risk, particularly among individuals with a high-PRS (OR_for lower coffee intake_ = 2.93, 95% CI = 1.71–5.04, *p* = 0.049; OR_for higher coffee intake_ = 2.15, 95%CI = 1.33–3.45, *p* = 0.049) [41].

#### 3.2.5. Meat and Fish

In the study conducted by Theodoropoulou et al., researchers explored the association between fish and meat consumption and the prevalence of cataracts. The results revealed a significant negative association between the consumption of fish and the incidence of all types of cataracts combined, as well as nuclear and PSC cataracts (OR_cataract overall_ = 0.69, *p* < 0.001; OR_nuclear cataract_ = 0.67, *p* = 0.001; OR_PSC cataract_ = 0.68, *p* = 0.034). In contrast, meat consumption was positively associated with all types of cataracts combined, as well as for nuclear, cortical, and PSC cataracts, separately (OR = 1.46, *p* = 0.001; OR = 1.53, *p* = 0.001; OR = 1.56, *p* = 0.05, and OR = 1.43, *p* = 0.021, respectively) [27].

### 3.3. ARC and Vitamins, Minerals and Carotenoids

#### 3.3.1. Antioxidants

In the meta-analysis conducted by Jiang et al., the researchers summarized findings on the association between dietary vitamins and carotenoids and ARC risk. This study involved a detailed analysis of 8 randomized clinical trials and 12 cohort studies from the United States, Australia, Finland, Canada, Puerto Rico, and Japan. The cohort studies analyzed by Jiang’s research team showed that the highest intake of vitamin A, compared to the lowest intake, was associated with a 19% decrease in the risk of developing ARC (RR = 0.81; 95%CI = 0.71–0.92; *p* = 0.001). Moreover, the dose–response analysis proved that an increase in vitamin A intake of 5 mg per day was linked to a 6% decrease in the risk of ARC (RR = 0.94, 95%CI = 0.90–0.98; *p* < 0.001) [32]. Similar findings were obtained in a meta-analysis conducted by Wang et al. in 2014. In that research, the highest consumption of vitamin A, compared to the lowest, was significantly associated with a 17% lower risk of cataracts overall (RR = 0.831; 95%CI = 0.757–0.913; I^2^ = 0.0%). However, the association was significant only in prospective studies (RR = 0.845; 95%CI = 0.759–0.940; I^2^ = 0.0%) [35]. Moreover, in the case of female participants, the consumption of vitamin A (OR_low_mid model1–3_ = [1.504, 1.416, 1.430]) was inversely associated with the prevalence of cataract incidence [42].

The pooled RR showed that the highest versus the lowest intake of vitamin E was associated with a 10% decrease in the risk of ARC (RR = 0.90; 95%CI = 0.80–1.00; *p* = 0.049) [32]. In the meta-analysis by Zhang et al., the research team evaluated eight papers with a total of 15,021 participants and, based on these data, determined the effect of vitamin E intake on the risk of cataracts in study subjects. The result showed that the highest dietary vitamin E intake, when compared to the lowest, was statistically significantly associated with the risk of ARC (RR = 0.73; 95%CI = 0.58–0.92; I^2^ = 69.1%; *p*_heterogeneity_ = 0.002). A dose–response analysis based on three studies found a non-linear association between vitamin E intake and ARC risk (*p*_for non-linearity_ = 0.0009). Starting from 7 mg of vitamin E per day, a statistically significant decreased risk of developing ARC was observed, with even higher results for the increasing vitamin E daily dosage (RR_7mg/d_ = 0.94, 95%CI = 0.90–0.97; RR_8mg/d_ = 0.89, 95%CI = 0.85–0.94; RR_9mg/d_ = 0.80, 95%CI = 0.74–0.88 and RR_10mg/d_ = 0.69, 95%CI = 0.59–0.80). Ten studies, encompassing 358,007 participants, did not show any significant association between supplementary vitamin E intake and the risk of ARC [33]. This is confirmed by Jiang’s study, in which the analysis of RCTs on vitamin E supplementation compared with the placebo exhibited no significant protective effect against ARC (RR = 0.97, 95%CI = 0.91–1.03, *p* = 0.262; RR = 0.99, 95%CI = 0.92–1.07, *p* = 0.820, respectively) [32]. In contrast, Zheng Selin’s Swedish cohort study proved, through a multivariable analysis, that vitamin E supplement use, compared to non-supplement-use, was associated with a higher risk of cataract (HR = 1.59, 95%CI = 2.12–2.26). Furthermore, the multivariable-adjusted hazard ratios for male participants who took other supplements in addition to vitamin E, compared to those calculated for non-supplement-users, were also associated with a higher risk of cataracts (HR = 1.02, 95%CI = 0.87–1.20) [36]. However, a comparison of the highest and the lowest combined dietary and supplementary vitamin E intake still proved an inverse association with ARC risk (RR = 0.86; 95%CI = 0.75–0.99; I^2^ = 47.1%; *p*_heterogeneity_ = 0.129) [33]. 

In a meta-analysis by Wei et al., the highest dietary vitamin C intake, compared to the lowest, was significantly associated with the risk of cataracts (RR_summary_ = 0.814, 95%CI = 0.707–0.938, I^2^ = 70.7%). Subgroup analyses by study design suggest that the association between the highest compared to the lowest consumption of vitamin C and cataract risk was significant in the case–control studies only (RR = 0.681, 95%CI = 0.549–0.845). In subgroup analyses of geographic locations, significant associations were noticed in both Asia (RR = 0.761, 95%CI = 0.592–0.979) and America (RR = 0.845, 95%CI = 0.730–0.978), but not in Europe. For the subgroup analysis by cataract type, a significant association was found only for nuclear cataract (RR = 0.618, 95%CI = 0.420–0.909, I^2^ = 81.8%) [34]. The stepwise multivariable regression used in Yonova-Doing’s cohort study analysis showed that vitamin C was significantly related to the nuclear dip score (NDS), both at baseline (β = −0.0002, SD = 6.3 × 10^−5^, *p* = 0.01) and during follow-up (β = −0.001, SD = 0.001, *p* = 0.03). It indicated that vitamin C inversely influenced the appearance of opalescence/opacifications of the lens, leading to a decrease in its pixel density, which was reflected by the NDS. Similarly to the previously mentioned study, the highest consumption of vitamin C, compared to the lowest, was associated with a 19% and 33% risk reduction at baseline and for cataract progression (RRR = 0.81, 95%CI = 0.68–0.96; RRR = 0.66, 95%CI = 0.47–0.91, respectively) [43]. 

The dose–response analysis conducted by Jiang showed that an increase in vitamin C consumption of 500 mg per day was significantly associated with an 18% decrease in the risk of ARC (RR = 0.82, 95%CI = 0.74–0.91, *p* < 0.001) [32]. In the Pastor-Valero study, increasing daily dietary intakes from 107 mg/d of vitamin C were also associated with a decreasing prevalence of cataracts (OR = 0.49, 95%CI = 0.27–0.92, *p* = 0.047) [26]. In terms of vitamin C supplementation, the multivariable analysis for only vitamin C supplement use, compared to non-supplement-use, was associated with a higher risk of cataracts (HR = 1.21, 95%CI = 1.04–1.41). The multivariable-adjusted hazard ratios for male participants who took other supplements in addition to vitamin C, in contrast to non-supplement-users, were also associated with a higher risk of cataracts (HR = 1.06, 95%CI = 0.93–1.20) [36].

The highest intake of β-carotene, compared to the lowest, was associated with a 10% decrease in the risk of ARC (RR = 0.90, 95%CI = 0.83–0.99, *p* = 0.023). In the dose–response meta-analysis, an increase in β-carotene consumption of 5 mg per day was significantly associated with an 8% decrease in the risk of ARC (RR = 0.92, 95%CI = 0.88–0.96, *p* < 0.001) [32]. In Wang’s study, the highest consumption of β-carotene, compared to the lowest, was also significantly associated with the risk of cataracts (RR = 0.937, 95%CI = 0.880–0.997, I^2^ = 1.2%). Subgroup analyses by study design suggested that the association between the highest compared to the lowest consumption of β-carotene and cataract risk was significant only in the prospective studies (RR = 0.872, 95%CI = 0.792–0.961, I^2^ = 0.0%). For the subgroup analysis by cataract type, a significant association was found only in PSC (RR = 0.713, 95%CI = 0.546–0.931, I^2^ = 0.0%) [35]. Moreover, β-carotene supplementation compared with the placebo had no significant positive effect on the risk of ARC [32].

The pooled RR showed that the highest versus the lowest consumption of lutein or zeaxanthin was associated with a 19% decrease in the risk of ARC (RR = 0.81, 95%CI = 0.75–0.89, *p* < 0.001). In the dose–response meta-analysis, an increase in lutein or zeaxanthin consumption of 10 mg per day was significantly associated with a 26% decrease in the risk of ARC (RR = 0.74, 95%CI = 0.67–0.80, *p* < 0.001) [32]. A lutein/zeaxanthin analysis conducted by Glaser et al. showed that, despite a protective trend in cataract prevalence, there was no significant association between its consumption and the risk of cortical and nuclear cataracts [37]. 

There was no significant association between total carotenoid, α-carotene, and β-cryptoxantin intake and the risk of ARC (RR = 0.83, 95%CI = 0.69–1.01, *p* = 0.059; RR= 0.96, 95%CI = 0.88–1.05, *p* = 0.369, I^2^ = 0.0%, *p*_heterogeneity_ = 0.818; RR = 0.95, 95%CI = 0.87–1.05, *p* = 0.332, respectively) [32].

#### 3.3.2. B-Group Vitamins

The association between consumption of B vitamins and the prevalence of cataract was assessed in 2015 by Glaser et al.. Their clinic-based, baseline cross-sectional and prospective cohort study showed that the highest dietary consumption of riboflavin (B2), as opposed to the lowest intake, was associated with a 22% (OR = 0.78, 95%CI = 0.63–0.97, *p* = 0.02) and 38% (OR = 0.62, 95%CI = 0.43–0.90, *p* = 0.01) lower prevalence of mild and moderate nuclear cataracts, respectively, as well as a 20% (OR = 0.80, 95%Cl = 0.65–0.99, *p* ≤ 0.05) lower prevalence of mild cortical cataracts. Also, the highest versus the lowest dietary consumption of vitamin B12 was associated with a 22% and 38% lower prevalence of mild and moderate nuclear cataracts and a 23% lower prevalence of mild cortical cataracts (OR = 0.78, 95%CI = 0.63–0.96, *p* = 0.02; OR = 0.62, 95%CI = 0.43–0.88, *p* = 0.01, and OR = 0.77, 95%CI = 0.63–0.95, *p* = 0.01, respectively). A 33% decrease in moderate nuclear cataract prevalence was found in the highest quintile of vitamin B6 consumption (OR = 0.67, 95%CI = 0.45–0.99, *p* ≤ 0.05) [37]. In Lee et al., the greater the consumption of vitamin B1 (OR_high_mid model 2_ = 0.685, *p* < 0.05; OR_high_mid model 3_ = 0.673, *p* < 0.05), the lower the prevalence of cataracts in male participants. In the case of female participants, the lower the consumption of vitamin B3 (OR_low_ mid unadjusted model_ = 1.382, *p* < 0.05), the higher the prevalence of cataract incidence. Moreover, an increase in vitamin B2 consumption indicated an increase in the prevalence of cataracts (OR_high_mid model 1,2,3_ = [1.364, 1.626, 1.639], *p* < 0.05) [42].

Glaser et al. reported that niacin was linked to a 31% lower risk of mild nuclear cataracts in Centrum^®^ non-users, and vitamin B12 was linked to a 44% lower risk of mild cortical cataracts (OR = 0.69, 95%CI = 0.52–0.92, *p* = 0.01 and OR = 0.56, 95%CI = 0.37–0.83, *p* = 0.01, respectively). Users of Centrum^®^ who reported consuming the most folate (B9) had a 61% higher risk of developing at least a mild PSC cataract compared to those who consumed the least amount of folate (OR = 1.61, 95%CI = 1.08–2.41, *p* = 0.02). Except for vitamin B9, there were no statistically significant negative correlations between the development of nuclear, cortical, or PSC cataracts and dietary consumption of any tested vitamins [37]. In Selin’s study cohort, participants who used vitamin B supplements, separately or combined with other supplements, had a significantly higher risk of cataract incidence than non-supplement-users (HR = 1.28, 95%CI = 1.12–1.43 and HR = 1.09, 95%CI = 1.02–1.17, respectively). In the youngest age group (under 60 years old), the use of B vitamins alone was related to a significantly increased risk of cataract development (HR = 1.88, 95%CI = 1.47–2.39) [38].

#### 3.3.3. Vitamin K

In a secondary analysis of an RCT conducted by Camacho-Barcia et al., researchers investigated the association between the consumption of vitamin K and the risk of ARC. The study showed that in the analysis adjusted for average energy intake, the highest consumption of dietary vitamin K_1_, compared to the lowest one was associated with a 29% lower risk of cataract surgery (HR = 0.71, 95%CI = 0.58–0.88, *p* = 0.002). A similar result was presented in sensitivity analysis, where the highest versus the lowest intake of dietary vitamin K_1_ was associated with a 25% decreased risk of cataract surgery (HR 3rd_tertile_ = 0.75, 95%CI = 0.60–0.92, *p* = 0.02) [39].

#### 3.3.4. Others

The study conducted by Yonova-Doing showed that manganese (β = −0.009, SD = 0.04, *p* = 0.03) was significantly related to NDS at baseline, implying that manganese had an inverse effect on the opalescence/opacification of the lens, resulting in a decrease in pixel density, which corresponded to the NDS. The highest consumption of manganese, compared to the lowest, was indeed associated with a 20% risk reduction at baseline (RRR = 0.80, 95%CI = 0.67–0.95) [43]. Another mineral that might affect the occurrence of cataracts is sodium. This assumption was confirmed by Bae et al.’s study, in which the prevalence of cataracts was significantly associated with high U[Na+]/Cr in participants over 50 (OR_for doubling U[Na+]/Cr_ = 1.13, 95%CI = 1.05–1.22, *p* < 0.01; OR_trends across quartiles_ = 1.11, 95%CI = 1.04–1.18, *p* < 0.01) [40]. Moreover, increased sodium intake could also indirectly influence the incidence of the disease by increasing the genetic risk of developing cataracts, especially in those with a high polygenetic risk score. As it was emphasized, a low-sodium diet might be beneficial in preventing ARC in the elderly population [41].

Other substances that have shown an effect on the incidence of cataracts include water (OR_high_mid unadjusted model_ = 0.726) in male participants, polyunsaturated fatty acids (OR _low_mid model1–3_ = [1.555, 2.026, 2.063]), and sugar (OR_low_ mid unadjusted model_ = 1.260) in female participants [42], as well as total fat (OR = 2.00, *p* < 0.001 for cataract overall; OR = 1.96, *p* = 0.001 for nuclear cataract; OR = 2.47, *p* = 0.016 for cortical; OR = 2.60, *p* = 0.001 for PSC), carbohydrates (OR = 0.39, *p* < 0.001 for cataract overall; OR = 0.40, *p* = 0.002 for nuclear cataract; OR = 0.27, *p* = 0.015 for cortical; OR = 0.30, *p* = 0.002 for PSC), and cholesterol (OR = 1.65, *p* < 0.001 for cataract overall, OR = 1.70, *p* = 0.001 for nuclear cataract, OR = 1.84, *p* = 0.029 for cortical, and OR = 1.92, *p* = 0.001 for PSC) in the combined male and female population [27].

## 4. Discussion

The information presented in this systematic review strongly suggests a relationship between diet and the incidence of ARC. Increased consumption of plant-based products (vegetables, fruits, legumes, and nuts), as well as skimmed yogurt, coffee, and fish appears to be particularly beneficial in reducing the prevalence of ARC [26,27,28,29,30,41]. Interestingly, the analysis of the studies indicates that dietary intake of vitamins is more advantageous than dietary supplements, especially for vitamin E, B9, and β-carotene [32,37]. Moreover, some research highlights the superior health benefits of a KBD and vegetarian diet, as well as dietary patterns rich in antioxidants and omega-3 fatty acids [21,22,24]. 

The lack of an effect of the Mediterranean diet on reducing the incidence of ARC is puzzling, especially when considering the components of this diet. After all, it is a nutrition model composed mainly of plant-based products rich in unsaturated fatty acids and antioxidants. Additionally, a study comparing the total antioxidant capacity (TAC) and total oxidant capacity (TOC) in cataract patients and a healthy control group revealed unfavorable levels in patients with ARC [50]. As demonstrated in the study by Pitsavos et al., closer adherence to the guidelines of the MedDiet was directly linked to an increase in TAC levels [51]. This fact, along with the already established negative association between a healthy dietary index, which includes the basic elements of the MedDiet, and the incidence of cataract, should all the more support the beneficial effect of the MedDiet on the prevalence of ARC [52]. Nevertheless, it is worth noting that there exist individual studies reporting both positive and negative associations between the Mediterranean-Style Dietary Pattern Score (MSDPS) and ARC [53,54].

It is possible that in the study conducted by Gracia-Layana et al., the control group exposed to a low-fat diet may not have facilitated the observation of the benefits of the Mediterranean diet per se, as the study was primarily focused on discovering correlations regarding its intake along with EVOO and nuts. The recommendations for a fat-restrictive diet were not coincidental, but based on the American Heart Association’s 2002 Guidelines, which advocated a wide range of vegetables, fruits, grains, low-fat or skimmed dairy products, fish, pulses, and white and lean meats. According to the literature reviewed, a vast majority of these recommended food products have shown beneficial effects on the occurrence or development of ARC [26,27,28,29,30,55]. The guidelines also advised limiting saturated fat intake to less than 10% of daily calories, keeping cholesterol intake under 300 mg/d, and reducing trans fatty acid consumption through the use of dietary substitutes. Additionally, they stressed the importance of reducing salt and alcohol consumption. It is important to note that the study may not have been designed to assess whether the Mediterranean diet could affect earlier stages of cataracts, as cataracts can develop slowly over time before requiring surgical removal [30,56].

An issue worth considering in future studies on the impact of diets on the course of certain diseases is the increasing globalization and the consequent homogenization of lifestyles and eating habits among populations. In the past, diets used to be shaped by the local availability of food products [57]. As highlighted in the research by Haile et al., changes in the MedDiet, including reduced caloric intake and expenditure, the inclusion of low-nutrient-dense foods, and modifications in food processing methods, have led to an elevated risk of deficiencies in certain vitamins, particularly folate and vitamins A and D, and inadequate intake of other nutrients [58]. Analyzing the above, it might be worth considering the benefits of using existing tools for assessing diet quality even before selecting and recommending the implementation of the chosen dietary regimen in the context of the patient’s diet therapy. Nevertheless, further research is needed to clarify the contribution of not only individual elements, but the entire Mediterranean diet’s input on the incidence or risk of ARC.

To date, there are no clear, uniform guidelines for slowing down and preventing ARC development. However, more and more studies are pointing to specific doses of vitamins that slow down the formation of cataracts or lower their occurrence. Expanded knowledge in this area could serve as an adjunct to already existing treatments and improve the lives of the elderly population in the future.

The presented review has some limitations and strengths that should be considered. A notable strength of this paper lies in the fact that it identifies gaps in the literature regarding uniform dietary recommendations for the elderly to mitigate or slow the progression of ARC. The selection of publications included in the presented systematic review was executed with the utmost care and meticulousness concerning assessment tools, which can also be considered an advantage of the study. All the authors have made efforts to ensure that the publication can be distinguished by its systematic approach to data analysis and the consistency of the presented results from multiple studies conducted in different parts of the world. The limitation of this paper is the fact that numerous studies assess the prevalence of individual nutritional factors but do not investigate their combined effect on ARC incidence. Despite the authors’ efforts to conduct a thorough manual search of databases, there remains a possibility that some crucial research may have been inadvertently omitted. Moreover, the geographic scope of this review, which primarily encompasses studies from Europe, Asia, North America, and Australia, with limited representation from Africa and South America, may influence the range of the obtained results.

## 5. Conclusions

Significant associations have been identified between the consumption of various products and ARC incidence. A careful analysis of these associations holds promise for the development of new and effective guidelines aimed at reducing the occurrence of ARC. In the long term, such recommendations could not only improve the visual acuity and, consequently, the quality of life of older individuals, but also influence the economic well-being of patients and their broader surroundings. The data collected in the above publication can significantly enhance our understanding of the impact of environmental factors on the development of ARC. This information has the potential to benefit not only ophthalmologists, but also primary-health-care practitioners, who can educate patients and raise their awareness of the impact of nutrition on the development of this disease. In addition, knowledge about nutrient deficiencies can serve diagnostic purposes and lead to improved health-promoting behaviors for disease prevention. The results of the studies in the current review are not only practical but also exploratory in nature, as they may reshape our comprehension of ARC pathogenesis. Through a multidisciplinary approach to this diagnostic and therapeutic challenge, it may be possible to establish an effective model for modern biomedical research. The findings from this review can contribute to providing better dietary recommendations for slowing down the progression of ARC, ultimately improving the quality of life for ARC patients.

## Figures and Tables

**Table 1:** Baseline characteristics of included studies.

First Author, Date (Quality)	Design	Location	Sample Characteristic	ARC Type	Key Findings
**ARC VS. DIETARY PATTERNS**					
**Mediterranean Diet (MedDiet)**					
García-Layana et al. [20], 2017 (good)	Randomized Controlled Trial (RCT)	Spain	5802 participants, from 55 to 80 years old	the type of cataract was not distinguished	The study did not reveal any significant difference between the MedDiet groups and the control group. Moreover, there was no decrease in the frequency of cataract surgery between the groups assigned to the MedDiet versus the control group. The observed rates (per 1000 person-years) were 16.9, 17.6, and 16.2 for the MedDiet + EVOO, MedDiet + Nuts, and control groups, respectively.
**Traditional Korean balanced diet (KBD)**					
Jee D, Park S. [21], 2021 (fair)	Cohort study	Korea	40,262 participants (1972 ARC patients and 38,290 healthy controls), from 51 to 77 years old	the type of cataract was not distinguished	The study showed that in the MS groups only KBD consumption was inversely associated with the risk of ARC. Moreover, participants with a high KBD diet in the MS group had significantly lower ORs for ARC risk compared to those with a low KBD diet. Irrespective of MS, neither the rice-based diet or the WD diet patterns affected ARC risk.
**Traditional and Dairy Products and Vegetable Dietary Patterns**					
Amini et al. [22], 2021 (good)	Case-control study	Iran	336 participants (168 patients with ARC and 168 healthy ones), from 40 to 80 years old	the type of cataract was not distinguished	The study demonstrated that dietary patterns referred to as “dairy products and vegetables” and “traditional” were inversely associated with cataracts. Participants who consumed a high “carbohydrate and simple sugar” diet had higher ORs for risk of cataracts compared to those with a low intake. There was no significant association between the “nuts, seeds and simple sugar” dietary pattern and cataracts.
**Vegetarian diet**					
Chiu et al. [23], 2020 (good)	Cohort study	Taiwan	4436 participants (1341 vegetarians and 3095 nonvegetarian), aged over 40	the type of cataract was not distinguished	The study found that a vegetarian diet was associated with a 20% reduced risk of cataracts. This association was more pronounced among individuals who were overweight.
**Nutrient patterns**					
Sedaghat et al. [24], 2017 (good)	Case-control study	Iran	295 participants (97 cataract patients and 198 matched controls), from 48 to 67 years old	the type of cataract was not distinguished	The study indicated that participants who consumed high “sodium pattern” and high “fatty acid pattern” diets had higher ORs for risk of cataract compared to subjects with low consumption. No significant association was observed between the “mixed pattern” diet and cataracts. Both “antioxidant pattern” and “omega-3 pattern” diets were negatively associated with the prevalence of cataracts.
**Pro-inflammatory diet**					
Shivappa et al. [25], 2017 (good)	Case-control study	Iran	295 participants (97 cataract cases and 198 healthy controls), from 48 to 67 years old	the type of cataract was not distinguished	The study showed a positive association between DII and cataract incidence as well as E-DII and the risk of cataracts.
**CATARACT VS. DIETARY PRODUCTS**					
**Fruit and vegetable**					
Pastor-Valero [26], 2013 (good)	Cross-sectional study	Spain	593 participants (274 men and 319 women) enrolled in the EUREYE study, aged over 65	the type of cataract was not distinguished	The study showed that a higher combined consumption of fruit and vegetables was significantly and negatively associated with the odds of cataract development. No association was found in separate analyses of the intake of fruit or vegetables and prevalence of cataracts.
Theodoropoulou et al. [27], 2014 (good)	Case–control study	Greece	628 participants (314 cataract cases and 314 controls), from 45 to 85 years old	nuclear, cortical, posterior subcapsular cataract	The study found that, unlike higher meat intake, increased consumption of plant-based products, such as vegetables, fruit, starchy products, legumes, nuts, and fish was significantly and negatively associated with the risk of cataracts. There was no significant association between the consumption of cereals, dairy products, added lipids and cataract risk.
Huang et al. [28], 2015 (good)	Meta-analysis	USA, Nigeria, Spain, Australia, Italy, Greece	9 articles involving 6464 cataract cases and 11,2447 participants	the type of cataract was not distinguished	The study showed that the highest vegetable intake, when compared to the lowest, was significantly and negatively associated with the risk of cataracts. In a stratified analysis of research design, relationships were also discovered in case–control studies and cohort studies. The highest vegetable consumption, as opposed to the lowest consumption, was significantly related to the risk of ARC in America and Europe, but not in other regions of the world.
Adachi et al. [29], 2021 (good)	Cohort study	Japan	71,720 participants (32,387 men and 39,333 women) from 45 to 74 years old	the type of cataract was not distinguished	The study revealed that male participants who consumed high rather than low amounts of total vegetables and cruciferous vegetables had a significantly lower prevalence of ARC. This association was more pronounced in men over 60 years old than in younger men. In female participants, the opposite results were observed.
**Dairy products**					
Camacho-Barcia et al. [30], 2019 (fair)	RCT	Spain	5860 participants, men from 55 to 80 years old and women from 60 to 80 years old, enrolled in the PREDIMED Study	the type of cataract was not distinguished	The study showed that participants with a higher intake of skimmed yoghurt had an inverse association with the risk of cataract surgery. The second tertile of total yogurt intake also indicated a negative association with cataract risk.
**Wholegrain and legume foods**					
Tan et al. [31], 2020 (good)	Cohort study	Australia	the baseline: 3654 participants; five years later: 2334 participants; ten years later: 1952 participants from BMES, aged over 49 years old	nuclear, cortical, posterior subcapsular cataract	The study did not find any significant associations between total wholegrain consumption or the consumption of any specific source of whole grain and any type of cataract incidence. There was no significant trend of a relationship between the highest and the lowest intake of legumes for PSC cataract incidence. No associations were found between pulse consumption and the incidence of cortical or nuclear cataract.
**CATARACT VS. VITAMINS, MINERALS AND CAROTENOIDS**					
**Antioxidants**					
Jiang et al. [32], 2019 (good)	Meta-analysis	USA, Australia, Finland, Canada, Puerto Rico, Japan,	8 RCTs and 12 cohort studies in total including 385,107 participants, aged over 40	nuclear, cortical, posterior subcapsular cataract	The study demonstrated that in RCTs, neither vitamin E or β-carotene supplements, when compared with the placebo, had any significant beneficial effect on the risk of ARC. Analyses of cohort studies showed that the highest levels of vitamins A and E, as compared with the lowest levels, were associated with a 19% and 10% decrease in the risk of ARC. An increase in vitamin A intake of 5 mg per day was associated with a 6% decrease in the risk of ARC. An increase in vitamin C consumption of 500 mg per day was significantly associated with an 18% decrease in the risk of ARC. The highest intake of β-carotene, as well as lutein or zeaxanthin, as opposed to the lowest intake, was associated with a 10% and 19% decrease in the risk of ARC. An increase in β-carotene consumption of 5 mg per day and in lutein or zeaxanthin consumption of 10 mg per day was significantly associated with an 8% and 26% decrease in the risk of ARC. No significant association was found between total carotenoid, α-carotene, and β-cryptoxantin intake and the risk of ARC.
Zhang et al. [33], 2015 (good)	Meta-analysis	USA, Australia, Finland, Sweden, Italy, Greece, Spain	18 articles (8 case-control studies, 5 RCTs, 4 cohort studies, 1 cross-sectional study) including 12,542 cataract cases	nuclear, cortical, posterior subcapsular cataract	The study showed that the highest compared to the lowest dietary vitamin E intake, as well as combined dietary and supplemental vitamin E intake, were statistically significantly associated with the risk of ARC. There was no significant association between solely supplemental vitamin E intake and risk of ARC. Dose-response analysis of dietary vitamin E intake and ARC risk found a statistically significant decreased risk of developing ARC with increasing dietary vitamin E intake from 7 mg/d.
Wei et al. [34], 2016 (good)	Meta-analysis	USA, Spain, Japan, Sweden, Italy, India, Australia	15 articles (11 prospective studies, 7 case-control studies, 1 RCT and 1 cross-sectional study) including 16,205 cataract cases	nuclear, cortical, posterior subcapsular cataract	The study revealed that the highest dietary vitamin C intake, as compared to the lowest intake of this vitamin, was significantly associated with the risk of cataracts. Subgroup analyses by study design suggest that the association between the highest compared to the lowest consumption of vitamin C and cataract risk was significant in the case-control studies only. In subgroup analyses based on geographic locations, significant associations were observed in both Asia and America, but not in Europe. In the subgroup analysis by cataract type, a significant association was found only in nuclear cataract.
Wang et al. [35], 2014 (good)	Meta-analysis	USA, Finland, Australia, Spain and Italy	- 12 articles with 17 studies including 7038 cataract cases for β-carotene intake - 6 articles with 11 studies including 5553 cataract cases for vitamin A intake	nuclear, cortical, posterior subcapsular cataract	The study showed that the highest consumption of β-carotene as well as vitamin A, as compared to the lowest consumption of these vitamins, was significantly associated with the risk of cataracts. For both β-carotene and vitamin A, the association was significant only in prospective studies. In the subgroup analysis by cataract type, a significant association was found only between β-carotene consumption and PSC. The study found no significant association between vitamin A intake and the risk of any specific type of cataract.
Zheng Selin et al. [36], 2013 (good)	Cohort study	Sweden	31,120 Swedish men, from 45 to 79 years old	the type of cataract was not distinguished	The study demonstrated that in the multivariable analysis, the use of only vitamin C and the use of only vitamin E supplements, when compared to non-supplement-use, were associated with a higher risk of cataracts. The multivariable-adjusted hazard ratios for male participants who took other supplements in addition to vitamin C or vitamin E, in contrast to non-supplement-users, were also associated with a higher risk of cataracts. Vitamin C or vitamin E from the diet were not associated with cataract risk.
**Vitamin B**					
Glaser et al. [37], 2015 (good)	Baseline cross-sectional and prospective cohort study	USA	3115 patients (6129 eyes) enrolled in the Age-Related Eye Disease Study, from 55 to 80 years old, followed up for mean of 9.6 years	nuclear, cortical, posterior subcapsular cataract	The study showed that the highest dietary consumption of vitamin B2, compared to the lowest, was associated with a 22% and 38% lower prevalence of mild and moderate nuclear cataracts and a 20% lower prevalence of mild cortical cataract, respectively. The highest dietary consumption of vitamin B12, compared to the lowest, was associated with a 22% and 38% lower prevalence of mild and moderate nuclear cataracts and a 23% lower prevalence of mild cortical cataract. A 33% decrease in the prevalence of moderate nuclear cataract was found in the highest quintile of vitamin B6 consumption. Niacin was linked to a 31% lower risk of mild nuclear cataract in Centrum^®^ non-users, and vitamin B12 was linked to a 44% lower risk of mild cortical cataract. Users of Centrum^®^ who reported consuming the most folate had a 61% higher chance of developing at least a mild PSC cataract compared to those who consumed the least folate.
Selin et al. [38], 2017 (good)	Cohort study	Sweden	13,757 women from the Swedish Mammography Cohort, from 48 to 83 years old and 22,823 men from the Cohort of Swedish Men, from 45 to 79 years old	the type of cataract was not distinguished	Participants who used supplements of vitamin B, either separately or in combination with other supplements, had a significantly higher risk of cataract incidence than non-supplement-users. In the youngest age group (under 60 years old), the use of B vitamins alone was related to a significantly increased risk of cataract incidence.
**Vitamin K**					
Camacho-Barcia et al. [39], 2017 (fair)	Secondary analysis of an RCT	Spain	5860 participants (2590 men and 3270 women), from 60 to 72 years old	the type of cataract was not distinguished	The study showed that the highest dietary intake of vitamin K_1_, when compared to the lowest, was associated with a 25% lower risk of cataract surgery.
**Others**					
Bae et al. [40], 2015 (fair)	Cross-sectional case-control study	Korea	12,693 participants, (2687 with cataracts aged from 51 years old and 10,006 controls) from KNHANES	nuclear, cortical, posterior subcapsular cataract	The study demonstrated that the prevalence of cataract incidence was significantly associated with high U[Na+] to Cr ratio in participants over 50.
Jee et al. [41], 2020 (fair)	Cohort study	Korea	41,067 participants (1972 with cataracts and 39,095 controls) aged over 50 years old	the type of cataract was not distinguished	The study revealed an association between sodium and coffee intake, as well as a Western-style diet and genetic risk in ARC development.
Lee et al. [42], 2022 (fair)	Cross-sectional study	Korea	5634 participants among whom 2137 had cataracts and 3497 did not, aged over 60 years old	the type of cataract was not distinguished	The study indicated that in male participants, higher consumption of both vitamin B1 and water was associated with a lower prevalence of cataracts. Among female participants, consumption of polyunsaturated fatty acids and vitamin A was inversely associated with the prevalence of cataracts. Likewise, lower consumption of vitamin B3 and sugar was linked to a higher prevalence of cataracts. Moreover, an increase in vitamin B2 consumption was correlated with an increase in the prevalence of cataracts.
Yonova-Doing et al. [43], 2016 (fair)	Cohort study	United Kingdom	324 female twin participants (151 monozygotic and 173 dizygotic twins), from 58 to 83 years old	nuclear cataract	The study showed that at baseline, NDS was significantly related to vitamin C and manganese. However, the association with cataract progression was only found for vitamin C. The highest consumption of vitamin C and manganese, as compared to the lowest, was associated with a 19% and 20% risk reduction at baseline, respectively. The highest consumption of vitamin C, compared to the lowest, was also associated with a 33% risk reduction in cataract progression. Only micronutrient supplements remained significant in the multivariate model, and their intake led to an 18% risk reduction in people within the highest tertile of nutrient intake, compared with the lowest.

## Data Availability

All information will be available upon an e-mail request to the corresponding author.

## Supplementary Materials

The following supporting information can be downloaded at: https://www.mdpi.com/article/10.3390/nu15214585/s1, Table S1: Quality assessment for included observational cohort and cross-sectional studies, Table S2: Quality assessment for included meta-analysis and systematic review, Table S3: Quality assessment for included case-control studies, Table S4: Quality assessment for included controlled intervention studies.
