# Supplementary material for: Influence of Diet, Dietary Products and Vitamins on Age-Related Cataract Incidence: A Systematic Review
Source: Nutrients. 2023 Oct 28;15(21):4585. doi: 10.3390/nu15214585 (PMC10650191; doi:10.3390/nu15214585)
Supplement: Supplementary file 1 [file nutrients-15-04585-s001.zip › nutrients-2660096-supplementary.pdf]

Supplementary Material

Influence of Diet, Dietary Products and Vitamins on Age-Related Cataract Incidence: A Systematic Review

Martyna Falkowska [1*], Maryla Młynarczyk [2], Zuzanna Micun [2], Joanna Konopińska [2] and Katarzyna Socha [1]

[1] Department of Bromatology, Faculty of Pharmacy with the Division of Laboratory Medicine, Medical University of Białystok, Mickiewicza 2D, 15-222 Białystok, Poland; martyna.falkowskaa@gmail.com (M.F.); katarzyna.socha@umb.edu.pl (K.S.)

[2] Department of Ophthalmology, Medical University of Białystok, M. Skłodowskiej-Curie 24a, 15-276 Białystok, Poland; mromaniuk2121@gmail.com (M.M.); z.micun@wp.pl (Z.M.); joannakonopinska@o2.pl (J.K.)

* Correspondence: martyna.falkowskaa@gmail.com

Table S1 Quality assessment for included observational cohort and cross-sectional studies

Criteria	Jee [21]	Chiu [23]	Pastor-Valero [26]	Adachi [29]	Tan [31]	Zheng Selin [36]	Glaser [37]	Selin [38]	Bae [40]	Jee [41]	Lee [42]	Yonova-Doing [43]
1. Was the research question or objective in this paper clearly stated?	yes	yes	yes	yes	yes	yes	yes	yes	yes	no	yes	yes
2. Was the study population clearly specified and defined?	yes	yes	yes	yes	yes	yes	yes	yes	yes	yes	yes	yes
3. Was the participation rate of eligible persons at least 50%?	n/r	yes	no	yes	no	yes	yes	no	no	n/r	no	no
4. Were all the subjects selected or recruited from the same or similar populations (including the same time period)? Were inclusion and exclusion criteria for being in the study prespecified and applied uniformly to all participants?	yes	yes	yes	yes	yes	yes	yes	yes	yes	yes	yes	yes
5. Was a sample size justification, power description, or variance and effect estimates provided?	no	no	yes	no	no	no	yes	no	no	no	yes	no
6. For the analyses in this paper, were the exposure of interest measured prior to the outcome being measured?	yes	yes	n/a	yes	yes	yes	yes	yes	n/a	yes	n/a	yes
7. Was the timeframe sufficient so that one could reasonably expect to see an association between exposure and outcome if it existed?	yes	yes	n/a	yes	yes	yes	yes	yes	n/a	yes	n/a	yes
8. For exposures that can vary in amount or level, did the study examine different levels of the exposure as related to the outcome?	yes	yes	yes	yes	yes	yes	yes	no	yes	yes	yes	yes

9. Were the exposure measures clearly defined, valid, reliable, and implemented consistently across all study participants?	yes	yes	yes	yes	yes	yes	yes	yes	yes	yes	yes	yes
10. Was the exposure assessed more than once over time?	n/r	no	no	yes	yes	no	yes	yes	no	no	no	no
11. Were the outcome measures clearly defined, valid, reliable, and implemented consistently across all study participants?	yes	yes	yes	yes	yes	yes	yes	yes	yes	c/d	no	yes
12. Were the outcome assessors blinded to the exposure status of participants?	no	no	no	no	yes	no	no	no	no	no	no	no
13. Was loss to follow-up after baseline 20% or less?	n/r	no	n/a	no	no	yes	n/r	yes	n/a	n/r	n/a	no
14. Were key potential confounding variables measured and adjusted statistically for their impact on the relationship between exposure and outcome?	yes	yes	yes	yes	yes	yes	yes	yes	yes	yes	yes	yes
**Quality category**	**fair**	**good**	**good**	**good**	**good**	**good**	**good**	**good**	**fair**	**fair**	**fair**	**fair**

Each question could be answered with yes, no or other (c/d, cannot determine; n/a, not applicable; n/r, not reported).

Table S2 Quality assessment for included meta-analysis and systematic review

**Criteria**	**Huang [28]**	**Jiang [32]**	**Zhang [33]**	**Wei [34]**	**Wang [35]**	**Hobbs**
1. Is the review based on a focused question that is adequately formulated and described?	yes	yes	yes	yes	yes	yes
2. Were eligibility criteria for included and excluded studies predefined and specified?	yes	yes	yes	yes	yes	no
3. Did the literature search strategy use a comprehensive, systematic approach?	yes	yes	yes	yes	yes	no
4. Were titles, abstracts, and full-text articles dually and independently reviewed for inclusion and exclusion to minimize bias?	yes	yes	yes	yes	yes	no
5. Was the quality of each included study rated independently by two or more reviewers using a standard method to appraise its internal validity?	no	yes	no	yes	no	no
6. Were the included studies listed along with important characteristics and results of each study?	yes	yes	yes	yes	yes	no
7. Was publication bias assessed?	yes	yes	yes	yes	yes	no
8. Was heterogeneity assessed? (This question applies only to meta-analyses.)	yes	yes	yes	yes	yes	n/a
**Quality category**	**good**	**good**	**good**	**good**	**good**	**poor**

Each question could be answered with yes, no or other (c/d, cannot determine; n/a, not applicable; n/r, not reported).

Table S3 Quality assessment for included case-control studies

Criteria	Amini [22]	Sedaghat [24]	Shivappa [25]	Theodoropoulou [27]
1. Was the research question or objective in this paper clearly stated and appropriate?	yes	yes	yes	yes
2. Was the study population clearly specified and defined?	yes	yes	yes	yes
3. Did the authors include a sample size justification?	yes	yes	no	no
4. Were controls selected or recruited from the same or similar population that gave rise to the cases (including the same timeframe)?	yes	yes	yes	yes
5. Were the definitions, inclusion and exclusion criteria, algorithms or processes used to identify or select cases and controls valid, reliable, and implemented consistently across all study participants?	yes	yes	yes	yes
6. Were the cases clearly defined and differentiated from controls?	yes	yes	yes	yes
7. If less than 100 percent of eligible cases and/or controls were selected for the study, were the cases and/or controls randomly selected from those eligible?	n/r	no	no	n/r
8. Was there use of concurrent controls?	yes	yes	yes	yes
9. Were the investigators able to confirm that the exposure/risk occurred prior to the development of the condition or event that defined a participant as a case?	yes	yes	yes	yes
10. Were the measures of exposure/risk clearly defined, valid, reliable, and implemented consistently (including the same time period) across all study participants?	yes	yes	yes	yes
11. Were the assessors of exposure/risk blinded to the case or control status of participants?	no	no	no	no
12. Were key potential confounding variables measured and adjusted statistically in the analyses? If matching was used, did the investigators account for matching during study analysis?	yes	yes	yes	yes
**Quality category**	**good**	**good**	**good**	**good**

Each question could be answered with yes, no or other (c/d, cannot determine; n/a, not applicable; n/r, not reported).

Table S4 Quality assessment for included controlled intervention studies

Criteria	García-Layana [20]	Camacho-Barcia [30]	Camacho-Barcia [39]
1. Was the study described as randomized, a randomized trial, a randomized clinical trial, or an RCT?	yes	yes	yes
2. Was the method of randomization adequate?	yes	c/d	c/d
3. Was the treatment allocation concealed?	yes	c/d	c/d
4. Were study participants and providers blinded to treatment group assignment?	yes	c/d	no
5. Were the people assessing the outcomes blinded to the participants' group assignments?	yes	c/d	c/d
6. Were the groups similar at baseline on important characteristics that could affect outcomes?	yes	yes	yes
7. Was the overall drop-out rate from the study at endpoint 20% or lower of the number allocated to treatment?	c/d	c/d	c/d
8. Was the differential drop-out rate (between treatment groups) at endpoint 15 percentage points or lower?	c/d	c/d	c/d
9. Was there high adherence to the intervention protocols for each treatment group?	yes	c/d	c/d
10. Were other interventions avoided or similar in the groups?	c/d	c/d	yes
11. Were outcomes assessed using valid and reliable measures, implemented consistently across all study participants?	yes	yes	yes
12. Did the authors report that the sample size was sufficiently large to be able to detect a difference in the main outcome between groups with at least 80% power?	c/d	no	no
13. Were outcomes reported or subgroups analyzed prespecified?	yes	yes	no
14. Were all randomized participants analyzed in the group to which they were originally assigned?	yes	yes	yes
**Quality category**	**fair**	**fair**	**fair**

Each question could be answered with yes, no or other (c/d, cannot determine; n/a, not applicable; n/r, not reported).
